# Artificial-Intelligence-Based Prediction of Clinical Events among Hemodialysis Patients Using Non-Contact Sensor Data

**DOI:** 10.3390/s18092833

**Published:** 2018-08-27

**Authors:** Saurabh Singh Thakur, Shabbir Syed Abdul, Hsiao-Yean (Shannon) Chiu, Ram Babu Roy, Po-Yu Huang, Shwetambara Malwade, Aldilas Achmad Nursetyo, Yu-Chuan (Jack) Li

**Affiliations:** 1 Rajendra Mishra School of Engineering Entrepreneurship, Indian Institute of Technology Kharagpur, Kharagpur 721302, India; saurabhjan07@gmail.com (S.S.T.); rambabu@see.iitkgp.ac.in (R.B.R.); 2 Graduate Institute of Biomedical Informatics, Taipei Medical University, Taipei 110, Taiwan; mail.aldilas@gmail.com (A.A.N.); jaak88@gmail.com (Y.-C.L.); 3 International Center for Health Information Technology (ICHIT), Taipei Medical University, Taipei 110, Taiwan; sv14kekade@tmu.edu.tw; 4 School of Nursing, College of Nursing, Taipei Medical University, Taipei 110, Taiwan; hychiu0315@tmu.edu.tw; 5 School of Medicine, Research Center of Sleep Medicine, Taipei Medical University, Taipei 110, Taiwan; 6 School of Medicine, Taipei Medical University, Taipei 110, Taiwan; b101100089@tmu.edu.tw; 7 TMU Research Center of Cancer Translational Medicine, Taipei Medical University, Taipei 110, Taiwan

**Keywords:** artificial intelligence, supervised machine learning, predictive analytics, hemodialysis, non-contact sensor, heart rate, respiration rate, heart rate variability

## Abstract

Non-contact sensors are gaining popularity in clinical settings to monitor the vital parameters of patients. In this study, we used a non-contact sensor device to monitor vital parameters like the heart rate, respiration rate, and heart rate variability of hemodialysis (HD) patients for a period of 23 weeks during their HD sessions. During these 23 weeks, a total number of 3237 HD sessions were observed. Out of 109 patients enrolled in the study, 78 patients reported clinical events such as muscle spasms, inpatient stays, emergency visits or even death during the study period. We analyzed the sensor data of these two groups of patients, namely an event and no-event group. We found a statistically significant difference in the heart rates, respiration rates, and some heart rate variability parameters among the two groups of patients when their means were compared using an independent sample t-test. We further developed a supervised machine-learning-based prediction model to predict event or no-event based on the sensor data and demographic information. A mean area under curve (ROC AUC) of 90.16% with 96.21% mean precision, and 88.47% mean recall was achieved. Our findings point towards the novel use of non-contact sensors in clinical settings to monitor the vital parameters of patients and the further development of early warning solutions using artificial intelligence (AI) for the prediction of clinical events. These models could assist healthcare professionals in taking decisions and designing better care plans for patients by early detecting changes to vital parameters.

## 1. Introduction

Hemodialysis (HD) has been one treatment of choice for renal replacement therapy among patients with possible renal dysfunction [1]. Hemodialysis uses an apparatus to filter blood that can be carried out either in a dialysis center or at home. By doing so, it replaces the natural function of the kidneys to remove waste and maintain blood pressure among renal failure patients [2]. Hemodialysis has proved to prolong survival of end-stage renal disease patients aged more than 75 with multiple comorbidities [3]. Typically, HD consists of three sessions per week and each session lasts around 4 h [4,5].

Despite its function in terms of prolonging patients’ life expectancy and increasing the quality of life, patients may undergo several clinical events during HD. Among them are infection, cardiovascular events, muscle spasm, and even death [6]. Research by Han [7] successfully predicted cardiovascular events using echocardiographic parameters, but not other non-cardiovascular and cerebrovascular events. Similarly, some studies have been conducted to predict cardiovascular events in liver transplant patients [8,9].

Ambient intelligence (AmI) is an evolving research area that attempts to bring intelligence to our environment and make it much sensitive to our day-to-day life [10]. Various technologies like sensors, sensor networks, pervasive computing, and artificial intelligence (AI) are used in AmI to make our environment more sensitive to living. AmI attempts to make our life easier and safer and is being deployed in many areas that affect human life. It is becoming more significant in healthcare in both clinical and non-clinical settings like telemedicine, home automation, health behavior informatics and patient monitoring [11,12,13,14,15,16,17]. For instance, AmI interacts with humans (e.g., patients) using sensors and might be able to predict upcoming events before they occur.

Research has been done in the past into which sensors can be used to monitor or detect some health conditions or parameters. However, the use of artificial intelligence using sensor data to predict an upcoming event is rarely found. Research has been carried out by Tereul et al. [18] utilizing an ultrasonic sensor to measure blood flow during dialysis sessions. Trebbels et al. [19] measured hematocrit levels by designing impedance-spectroscopy-based sensors for dialysis apparatus. Yi-Chun Du et al. [20] proposed a wearable device to monitor blood leakage during HD using an array sensing patch. However, these studies did not embed the technology with an artificial intelligence feature. Artificial intelligence has been used to improve anemia management during dialysis treatment [21].

Methods or devices using physical contact with patients during their clinical care do not offer round-the-clock monitoring of their vital signs. On the other hand, data on vital signs, like blood pressure, heart rate, respiration rate, and body temperature are very important factors when a decision is being taken by the physician and healthcare professional. Nowadays, non-contact sensor devices are available that offer round-the-clock monitoring of the various vital signs of patients [22,23]. Further, this data can also be useful in predicting clinical events in patients for several clinical processes like HD, liver transplants, kidney transplants, etc. Various studies predicting cardiovascular events in liver transplant patients have been carried out in the past using contact or non-contact sensor data. However, there have been very few studies conducted to predict clinical events in HD patients using non-contact sensors.

In this study, a prediction model was developed based on supervised machine learning (ML) algorithms to predict clinical events during dialysis sessions. We used the data from a non-contact sensor device [24] that records vital signs like the heart rate, respiration rate, and heart rate variability. In the following sections, we present our methodology, results, discussion, limitations and conclusion.

## 2. Materials and Methods

### 2.1. Study Details

In this observational study, we included 109 patients who were undergoing HD at Taipei Medical University (TMU) hospital, Taipei, Taiwan. To conduct this study, ethical clearance was taken from the Joint Institutional Review Board (JIRB) of Taipei Medical University, Taipei, Taiwan (JIRB No. N201512031). During the period of HD, some patients experienced clinical events of certain types and a few patients did not report any clinical event. A clinical event can be defined as any medical problem experienced and reported by the patient. The clinical events suffered by the patients and considered in this study were sudden death (SD), emergency visit (ER), muscle spasm (MS), inpatient (IP), emergency visit and inpatient (ERIP).

### 2.2. Data Collection

Patient data were collected using a piezo-electric non-contact sensor system that measured respiration rate (RR), heart rate (HR), and body movement data (MD). These vital parameters of the patients were captured during the HD sessions for a period of 23 weeks from March 2016 to August 2016. The sensor unit was placed under the mattress and had no direct contact with the patient. The sensor system used in this study was developed by EarlySense Ltd., Ramat Gan, Israel [24]. When this sensor is kept under the mattress, a force is applied to the sensor that comes from three sources. These sources are gross body movement, chest wall movement, and recoil of the body due to the cardio-ballistic effect. The latter two are related to respiration effort and stroke volume. The signals generated by the sensor can be separated into motion, respiration and ballistocardiogram (BCG) waveforms, from which MD, RR and HR can be obtained, respectively [24]. The sensor system for patient monitoring in hospital settings has been validated and used in many clinical studies [22,25,26,27]. Similarly, the heart rate variability parameters like high frequency (HF), low frequency (LF), the ratio of HF–LF (HF/LF), and very low frequency (VLF) were also obtained from the raw BCG waveforms [28,29]. The heart rate variability (HRV) parameters including HR, RR, and MD were processed from the raw data and provided to us by a data scientist from EarlySense Ltd. The final dataset we received contained the mean of every 30 s for HR, RR, MD, and all the HRV parameters along with the date and patient ID (without revealing the personal identity of the patient). The demographic data like gender, age, height, and weight of the patients was also obtained during the study using a predesigned form.

### 2.3. Data Cleaning and Feature Extraction

There were some errors, missing values, and duplicate data files in the raw dataset. The raw data was cleaned to remove all the errors, missing values, and duplicate data files. This was done programmatically in the Python programming language and using the library *pandas*. The errors were the presence of the values 0 and −1 or missing values in some data fields. The entire row was deleted if it had either of these errors in any of its data fields. In the case of duplicate data files for the same HD session, the file which had the lesser number of data readings was ignored and that with data readings was selected. We had the following data variables after data cleaning and arranging the data from different files into a single data file:

Patient ID, date of session, RR, MD, HR, and HRV parameters like HF, LF, HF/LF, VLF, and (VLF+LF)/HF. We also had demographic details of the patients like the gender, age, height, and weight of the patients.

Since HRV parameters will be accurate only when the patient is relaxed and stable, we considered only those data samples in the analysis when the patient was in a stable and non-moving condition [30]. Therefore, we utilized the body movement data and further shortlisted only those data samples where the value of MD was less than a threshold of 30 amplitudes. Further, we extracted data samples for the vital parameters from the first five minutes (FFM) and last five minutes (LFM) of HD sessions. The reason for extracting FFM and LFM was to see the change in the vital parameters as the HD session progresses. The FFM data was extracted from the early period of the HD session and LFM was extracted from the end period of the session, since the short-term recording (5 min) of HRV is sufficient for monitoring the autonomic nervous system [31]. Therefore, in this study, instead of considering HRV and other recorded vital parameters for a complete length of the HD session, only 5 min of data samples from the beginning and end of the HD session were considered. We also extracted the total number of HD sessions a patient had attended and the total number of clinical events reported by a patient. In addition, the body mass index (BMI) of each patient was calculated from the height, weight and gender data information of the patient. Finally, we had the following variables:

Patient ID, Number of Sessions, Number of Events, Time_FFM, HR_FFM, RR_FFM, MD_FFM, HF_FFM, LF_FFM, HF/LF_FFM, VLF_FFM, (VLF+LF)/HF_FFM, Time_LFM, HR_LFM, RR_LFM, MD_LFM, HF_LFM, LF_LFM, HF/LF_LFM, VLF_LFM, (VLF+LF)/HF_LFM, Gender, Age, Weight, Height, BMI, and Class. Here, the variable “Class” represents the two categories of patients, one in which the patient had reported any of the five mentioned clinical events and the other in which patient had not reported any clinical event. We selected a subset of features from the above-mentioned features as input variables. The backward elimination method was used to select an optimal subset of features.

### 2.4. Model Development

**Figure 1:**
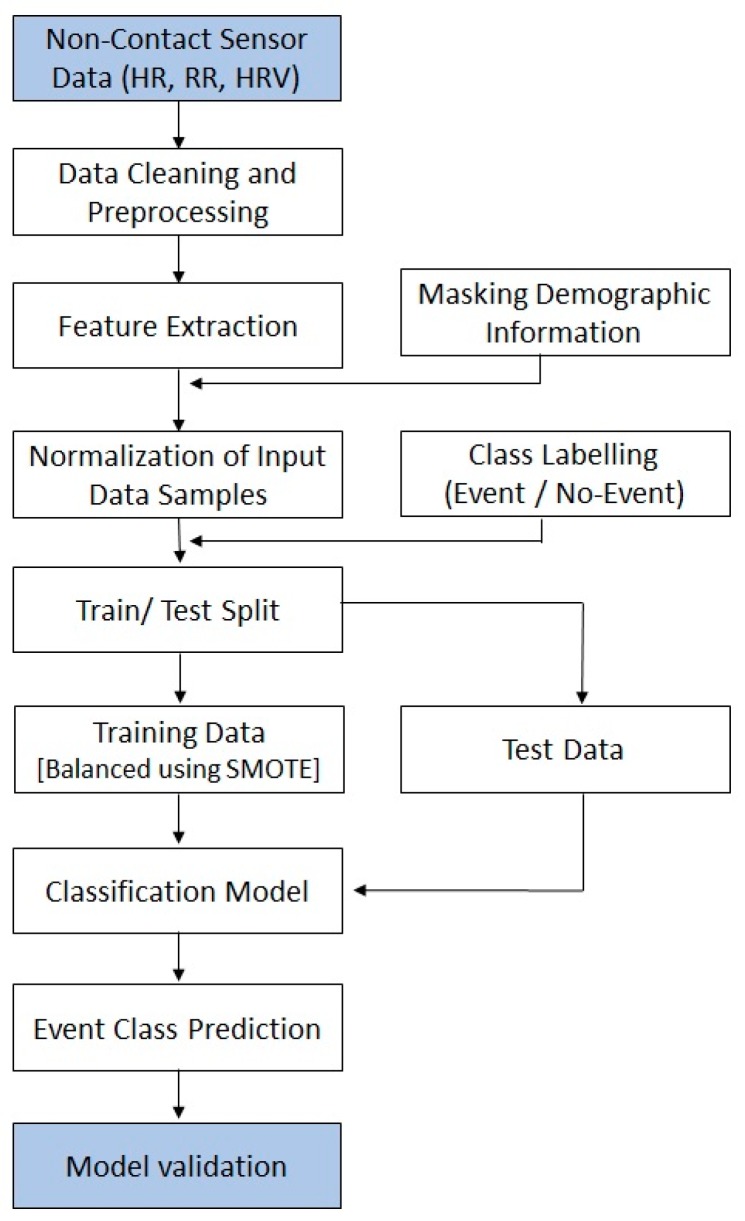
Prediction model for event class prediction. HR: Heart Rate; RR: Respiration Rate; HRV: Heart Rate Variability; SMOTE: Synthetic Minority Oversampling Technique.

In this work, we developed three predictive models. In each model, we tested five supervised machine-learning-based classification algorithms. We will call these classification algorithms classifiers. The classifiers used in each model were logistic regression (LR) [32,33], k-nearest neighbor (kNN) [34,35], adaptive boosting (AdaBoost) [36,37], random forest (RF) [38,39], and support vector machine (SVM) [40]. The results of the performance of the various classifiers are reported in the next section. To develop these classifiers, we used the Python distribution *Anaconda* of version 5.1.0 and various libraries of *scikit-learn 0.19.1* [41]. Each parameter recorded was sampled at the period of 30 s i.e., and for each 30 s we have one reading for all the vital parameters recorded by sensor. The basic difference between the three models being discussed in this study is the number of samples selected in each model. In model 1, we considered all the data samples selected for FFM and LFM. For each five-minute data we had 10 readings of all the vital parameters at the rate of 30 s. In model 2, we considered one sample for each HD session from each patient. The considered sample was the mean of all samples of FFM and LFM for that HD session. In this model, we also added more input features. These features are the variance of FFM and LFM of all the vital parameters. In model 3, we considered only one data sample corresponding to each patient by further taking out the mean of all the input features present in model 2. A block diagram of the predictive model is shown in Figure 1.

The models developed in this study are being trained and tested on two datasets. Since, the number of HD sessions undergone by each patient was different, there were chances of overfitting the classifiers. As some patients had a greater number of HD sessions, they had more data samples in comparison with other patients. Therefore, we selected data samples from 15 recent HD sessions for each patient. In this process, data samples of 105 patients who had undergone at least 15 HD sessions were selected and we discarded the data samples of the remaining four patients. Of these 105 patients, 76 had reported an event during the study period and 29 had not reported any event. In this way, we separated two datasets, one with data samples from all the HD sessions for all the patients and one with data samples taken only from the most-recent 15 HD sessions.

The dataset in which data samples from all HD sessions was considered had 109 patients who underwent a total number of 3237 HD sessions. Therefore, for this dataset, model 1 had a total number of 32,370 data samples. Model 2 had 3237 data samples and model 3 had 109 data samples. The data set in which data samples from the 15 recent HD sessions were selected had a total number of 105 patients with 1575 HD sessions. Therefore, model 1 had 15,750 data samples, model 2 had 1575 data samples and model 3 had 105 data samples.

### 2.5. Model Validation

There is unequal representation of the two classes because the number of patients from the event class is much higher than the number of patients from the no event class. To overcome this imbalance in the data we used the synthetic minority over-sampling technique (SMOTE) [42]. In this technique, the minority class is over-sampled to balance the representation of both the classes. This is done by “taking each minority class data point and introducing synthetic examples along the line segments joining any or all of the k-minority class nearest neighbors” [43]. The process is repeated until the representation of both classes becomes approximately equal. This approach can be implemented in several open-source software (e.g., the R Programming Language, Python, and Weka). More information on SMOTE is available in reference [42]. In this study, SMOTE was applied only to the training dataset and the testing dataset was left unchanged. To validate the model, we used the stratified *k*-fold cross-validation method [44]. In stratified cross-validation, the folds are selected such that the percentage of samples is preserved for each class. In each fold of the validation, the testing dataset was first balanced using SMOTE and then the classifier was trained.

In this study, the precision, recall, accuracy and receiver operating characteristic area under curve (ROC AUC) were used as evaluation measures to evaluate the performance of the various classifiers being developed [45]. A brief description of all these measures are given below:

#### 2.5.1. Precision

Precision (P) is the fraction of true positives (TP) predicted to the total predicted positives i.e., true positives plus false positives (FP). In some scenarios, it is also called as confidence. Precision is defined as:

(1) $$P=\frac{\mathrm{TP}}{\mathrm{TP}+\mathrm{FP}}.$$

#### 2.5.2. Recall

The recall is the fraction of TP predicted from the total of real positives i.e., true positive plus false negatives. It is also sometimes referred to as sensitivity. The recall is an important measure in the context of medical or clinical studies because it identifies all real positive cases. It is also important in ROC analysis in which it is referred to as the true positive rate. It is defined as:

(2) $$R=\frac{\mathrm{TP}}{\mathrm{TP}+\mathrm{FN}}.$$

#### 2.5.3. Accuracy

Accuracy is one of the most intuitive and basic performance measures for any ML model. It is not a good criterion by which to evaluate any model when the dataset is imbalanced. However, it is a good measure for a balanced dataset where all the classes to be classified are equally represented. It can be defined as:

(3) $$\mathrm{Accuracy}=\frac{\mathrm{TP}+\mathrm{TN}}{\mathrm{TP}+\mathrm{FP}+\mathrm{TN}+\mathrm{FN}}.$$

#### 2.5.4. Receiver Operating Characteristic

This is one of the most important and widely accepted evaluation criteria. It compares the true positive rate (TPR) and the false positive rate (FPR). It is created by plotting the TPR against the FPR. TPR i.e., sensitivity or recall is already defined above and FPR can be defined as follows:

(4) $$\mathrm{FPR}=\frac{\mathrm{FP}}{\mathrm{FP}+\mathrm{TN}}.$$

#### 2.5.5. Descriptive Analyses and Independent *t*-Test

We used SPSS 22.0 (IBM, Armonk, NY, USA) to carry out basic statistical analyses of the data. Descriptive analyses were used to examine the baseline characteristics. Differences in the HR, RR and HRV parameters between the event and no-event groups were examined using the independent samples *t*-test. The P value is described in terms of rejecting the null hypothesis when it is true. The null hypothesis is usually a hypothesis of no difference (i.e., no difference in a variable among the two groups). The term alpha refers to a pre-chosen probability and the term “*p* value” is the calculated probability. The choice of the significance level at which the null hypothesis can be rejected is subjective. Conventionally, the 5%, 1% and 0.1% (*p* < 0.05, 0.01 and 0.001) levels have been used.

In this study, the statistical significance (alpha) was set at *p* < 0.05. We selected the level of significance i.e., alpha = 0.05 so that the probability of making a wrong decision is at most 5% when the null hypothesis is true and also to balance the tradeoff between type 1 and type 2 errors. It also keeps the type 2 errors within acceptable limit and does not reject a null hypothesis (even if there is a significant difference when it is false). A type I error is the false rejection of the null hypothesis and a type II error is the false acceptance of the null hypothesis. While testing the hypothesis on the difference in mean for the event and no-event samples, we had two samples (for the event and no-event group) with different sizes. However, the differences in sample size have been accounted for in the computation of t-statistics (that is, in the estimation of the standard error of difference) for the hypothesis testing.

### 2.6. Sample Size

Since this study is in the medical domain, we have inherent limitations on our sample size due to problems associated with data collection from patients. We validated the sample size requirements for this study statistically. We determined the sample size requirement based on our assumption of a 50% prevalence rate and an error margin of 10% at the 95% confidence level. The required sample size turned out to be, *n* = 96. We used the standard formula for sample size determination used in cross-sectional studies [46].

## 3. Results

The baseline characteristics of the data used in this study are presented in Table 1. The values are presented for both the datasets considered in the study, namely the one that had 109 patients with data for all HD sessions and the other which had 105 patients with data from the most recent 15 HD sessions. Of 109 patients, 78 patients suffered from clinical event(s) and 31 patients did not report any clinical event during the whole period of HD. The total number of clinical events reported by patients during the study period was 166 and the frequency of events reported per patient ranged from 1–9. The distribution of different types of events is presented in Table 2. Patients were 30–89 years old with mean age 66.3 and standard deviation (std.) ± 12.2. There were 58 (53.2%) male and 51 (46.8%) female patients in the study. The BMI of patients ranged from 17.4 to 42.3, with a mean of 24.1 and std. ± 12.2.

A descriptive analysis was carried out in which the mean of all the variables was compared between the event group and the no-event group and are shown in Table 3. This was carried out on data samples selected from 15 recent HD sessions. A total number of *n* = 11,400 data samples were from the event group of patients and *n* = 4350 were from the no-event group of patients. We found that the mean heart rate, respiration rate and some HRV parameters were significantly different between the two groups. The mean heart rate and respiration rate of both the first five minutes and the last five minutes were different in the two groups with *p* < 0.0001. The mean heart rate of the first five minutes and last five minutes in the event group were found to be higher than those in the non-event group (FFM: 75.58 vs. 70.56 and LFM: 75.85 vs 70.61). Similarly, the respiration rate was also observed to be different among the two groups. It is also higher in the event group as compared to the no-event group (FFM: 17.80 vs. 16.48 and LFM: 17.49 vs. 16.11).

A statistically significant difference was found in the mean values of the vital parameters among event and no-event patients. Therefore, it became the basis for proceeding with further data analytics. As mentioned in the previous section, three predictive models were developed to predict the clinical event. The models were validated using the stratified k-fold cross validation method where *k* = 10. In model 1 and model 2, the dataset was divided into a training and validation set in each fold at the observation level, and at the patient level in model 3. In models 1 and 2, a single patient had many data samples, i.e., observations, therefore data splitting in these models occurred at observation level. However, each patient had a single corresponding data sample in model 3, therefore the data splitting occurred at the patient level in this model.

In each model, we tried and tested various classification methods like kNN, AdaBoost, SVM, RF and logistic regression (LR). When these classifiers were trained using the dataset in which data samples from all the HD sessions of patients were considered, the performance of the AdaBoost classifier which is an ensemble ML technique, was found to be better in all the models. kNN and SVM also performed well in model 1 and model 2. In model 1, a mean accuracy of 89.48% was achieved using kNN with 96.21% mean precision and 88.47% mean recall. The mean area under curve (AUC) was 90.16% in kNN classifier of model 1. Similarily, in AdaBoost the mean accuracy of 83.81% was achieved with mean precision, recall and AUC at 94.07%, 82.09%, and 83.81% respectively. In model 1, the mean accuracy of SVM was 76.94% with mean precision and mean recall of 90.19% and 75.28%, respectively, whereas in random forest and logistic regression the classifier recall was 62.84% and 59.76%, respectively. A decent accuracy with high precision, recall and area under ROC curve were obtained through the AdaBoost, kNN and SVM classifiers in model 1 and AdaBoost further performed better in model 2 and model 3 among other classifiers.

**Figure 2:**
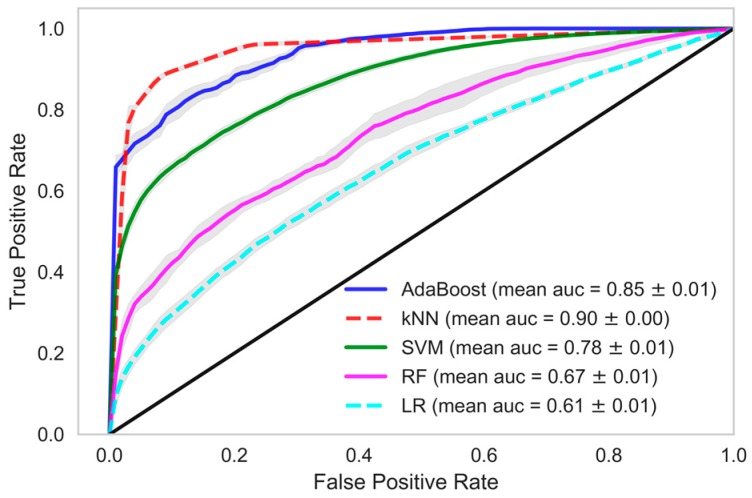
Receiver Operating Characteristics plot showing mean area under curve of all the classifiers of Model-1 when all HD sessions were considered.

**Figure 3:**
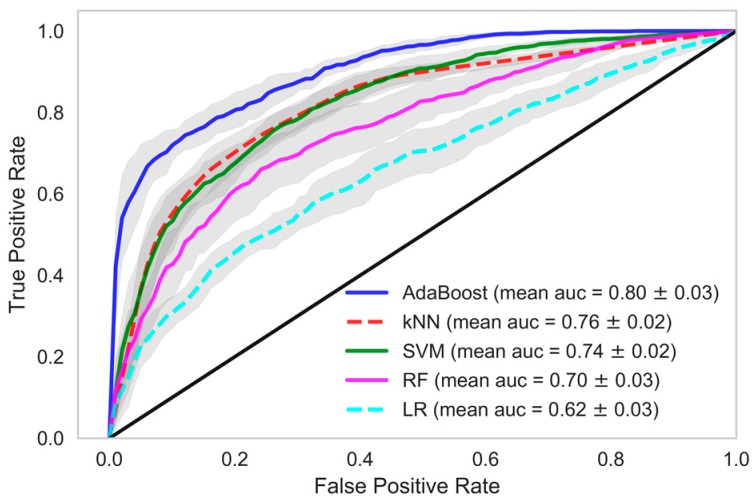
Receiver Operating Characteristics plot showing mean area under curve of all the classifiers of Model-2 when all HD sessions were considered.

**Figure 4:**
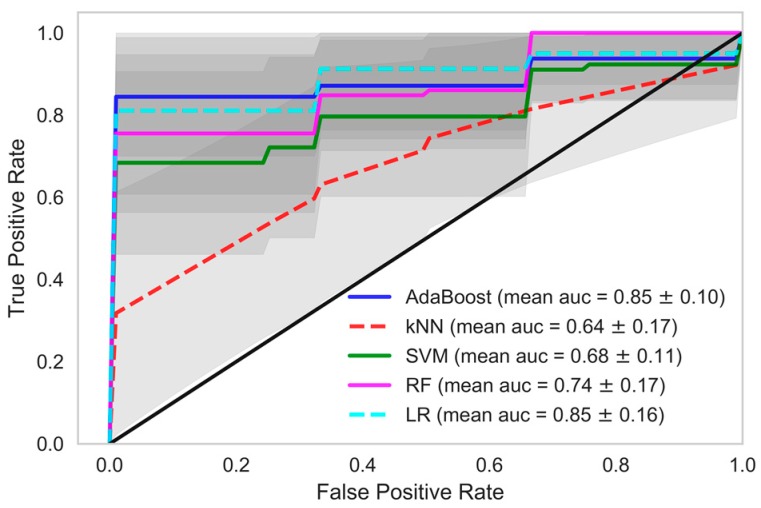
Receiver Operating Characteristics plot showing mean area under curve of all the classifiers of Model 3 when all HD sessions were considered.

**Figure 5:**
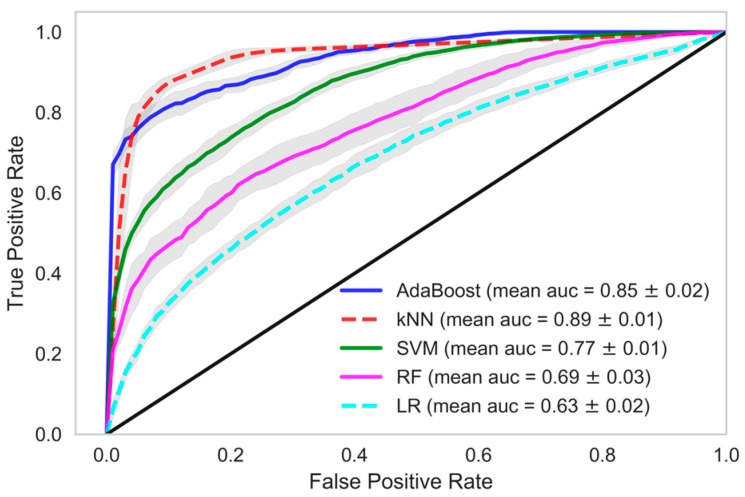
Receiver Operating Characteristics plot showing mean area under curve of all the classifiers of Model 1 when the 15 recent HD sessions were considered.

**Figure 6:**
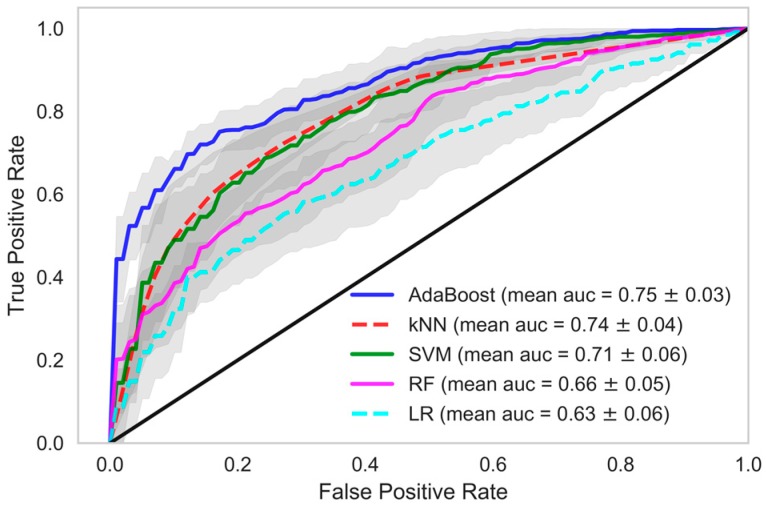
Receiver Operating Characteristics plot showing mean area under curve of all the classifiers of Model 2 when the 15 recent HD sessions were considered.

**Figure 7:**
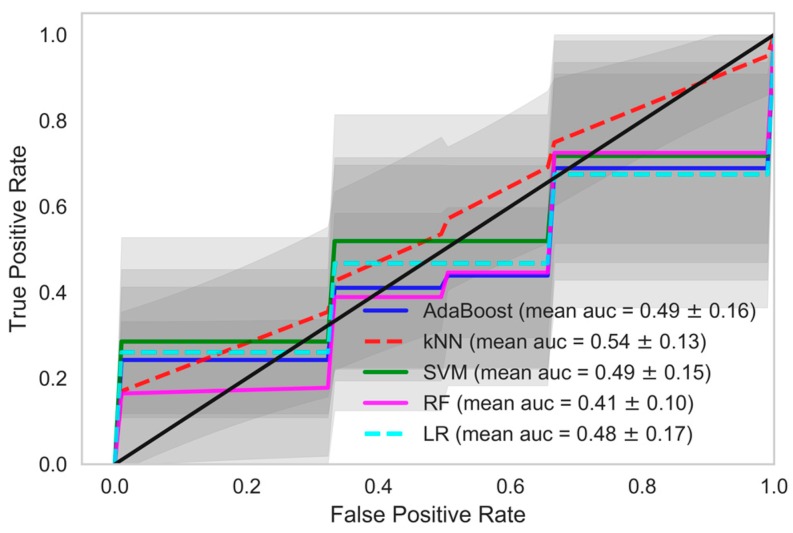
Receiver Operating Characteristics plot showing mean area under curve of all the classifiers of Model 3 when the 15 recent HD sessions were considered.

When we used data samples from recent 15 HD sessions to train the models, the performance of the classifiers was in line with those reported using data samples from all HD sessions. The mean accuracy of the kNN classifier was observed to be 87.95% with 89.01% AUC, 96.35% precision and 86.64% recall. The performance of the AdaBoost classifier in model 1, when this dataset was used, was also similar. A mean AUC of 85.38% with 81.86% recall and 95.09% precision was observed. The comparison of all the classifiers under each model considering various evaluation criteria was shown in Table 4, when data samples from all the HD sessions were considered in developing the model. In Table 5, evaluations of all the classifiers are shown when the models were developed using only the data samples from the 15 recent HD sessions. The ROC AUC plots of all the models depicting the mean area under the curve is presented in Figure 2, Figure 3, Figure 4, Figure 5, Figure 6 and Figure 7. The ROC AUC plots of each classifier under each model when the data samples from all the HD sessions were considered are shown in Figure 2, Figure 3 and Figure 4, respectively, and the ROC AUC plots when data samples from the 15 recent HD session were considered are shown in Figure 5, Figure 6 and Figure 7, respectively.

## 4. Discussion

In this study, we presented the novel use of non-contact sensor data in clinical settings during the process of HD of 109 patients. Hemodialysis is a procedure to purify the blood when the kidneys stop functioning normally. Hemodialysis may cause certain complications like cardiovascular, non-cardiovascular, and cerebrovascular issues, and even sudden death. During the 3237 HD sessions and 23 weeks of this study, we found that 78 patients reported 166 episodes of clinical events. We compared the differences among the group of patients who reported clinical events to those who did not report any clinical event. We found a statistically significant difference in the heart rates and respiration rates (*p* < 0.0001) recorded by the non-contact sensors among the two groups of patients. Various HRV parameters also showed significant differences, like high frequency and low frequency. The heart rate in both the first five minutes and last five minutes was found to be higher in the event group of patients compared to the no-event group of patients by an almost five-point basis. A similar pattern was observed for the respiration rate. Our findings suggest that an increased heart rate and respiration rate indicates the unwellness of the patient and that some kind of clinical event may happen in the near future that needs attention and care in advance.

It is further observed that the mean heart rate values of the last five minutes (HR_LFM) were slightly higher than the heart rate values for the first five minutes (HR_FFM). Various studies conducted in the past also point towards an increase in heart rate during HD [47,48,49,50]. This phenomenon is observed across both groups of patients. It is generally observed that the heart rate increases during HD because of the removal of excess body water, which triggers a significant increase in the heart rate. This finding is consistent with those reported in the literature. The mean values of all vital parameters are shown in Table 3. The basic statistical analysis shows differences in the values of most of the vital parameters among the two groups of patients that were also found to be statistically significant.

The performance of the developed predictive models was also in line with the statistical findings. We obtained very high accuracy for our ML-based predictive models. In this study, we demonstrated the use of non-contact sensors to monitor the vital parameters of patients during HD and in the early prediction of any clinical event that may occur during the period of hemodialysis-based treatment. The results of our study show the possibility of accurately and precisely predicting chances in a clinical event. Although we were dealing with a smaller dataset, the model we developed still performed extremely well. If we evaluate our dataset in terms of the frequency of data points, we had a reasonable number of data samples, namely 32,370. However, if we evaluate our dataset in terms of individual patients, we had only 109 data points, which is very few to train a ML model. Model 1 exploited the large frequency of our dataset, whereas model 3 relied on the individual patients. In model 1, we achieved 90.16% ROC with a recall rate of 88.47% with a kNN classifier and ROC of 84.97%, with a recall of 82.09% in the AdaBoost classifier. In model 2, we achieved 80.36% ROC with a recall rate of 80.73% with an AdaBoost classifier. Recall or sensitivity analysis was an important measure in the context of our study. It tells us the rate of predicting true positives. A high recall rate was achieved in this study, which validates the practicality of an early warning system based predictive analytics using non-contact sensor data in hospital settings. This type of system can alert the healthcare provider, physicians, and healthcare professionals so that they provide extra care to the patient and the patient can be saved from any forthcoming life-threatening clinical events.

In our model, we selected a subset of variables from the available variables. We used heart rate and respiration rate, two features that had HF and LF from the HRV along with age, gender and BMI as input features in our models. The rest of the available variables were not considered as input features for the models. The notion behind selecting HR and RR is that they are the two main vital parameters used for continuous patient monitoring. The objective of this study was to understand whether we can make early predictions of clinical events by monitoring these vital parameters. The HRV parameters are basically variables derived from the heart rate. HRV is related to the autonomic nervous system. High frequency is linked to the parasympathetic nervous system (PSNS) and LF is linked to the sympathetic nervous system (SNS), as reported in the literature [51]. The other HRV variables were not considered as input features because they did not have enough influence on the outcome of the classifiers. Therefore, we considered these variables as important markers for the prediction of the physiological health of patients. Further, there are various methods for deciding the best subset of features [52] reported in the literature, namely the filter method, wrapper method, and embedded method. We used the backward elimination technique of the wrapper method for feature selection, which showed optimum performance for the predictive models developed in this study.

We found that under real circumstances the use of non-contact sensors is highly beneficial in two ways. Firstly, it provides the continuous monitoring of the vital parameters of the patient during the HD session. It can help the physician or the care provider to take a decision in providing better care and comfort to the patients. Secondly, this data can be used for analyses and the prediction of any clinical emergency. It could predict event occurrences based on the increased heart rate and respiration rate during HD. Although it would be early to say that the proposed supervised ML-based predictive model could be used in real life, on the basis of the obtained results we suggest that more such studies are conducted using non-contact sensors in clinical settings. These proposed studies could validate the findings of this study and further be useful in the development of a more robust predictive application to predict clinical events in advance.

## 5. Limitations of This Study

We had certain limitations in this study in terms of the clinical variables available for analysis. We had only three critical parameters in our dataset to build our model, i.e., heart rate, respiration rate, and HRV parameters. The inclusion of other vital parameters like blood pressure and patient’s medical history could add more weight to such predictive modeling approaches. One of the major issues observed in HD is the sudden death of patients, including various other events like ER visits or inpatient stays due to cardiovascular problems, infection, renal problems, etc. During this study, six people suffered sudden death. It was one of our objectives to develop a multi-class prediction model that is able to predict chances of different events occurring in advance. We had details of the medical issues reported during the clinical event in the form of International Classification of Diseases (ICD) codes, but the number of cases reported was very low. Therefore, in this study we limited our analysis to a binary classification model instead of building a multi-class classification model.

## 6. Conclusions

The initial findings and the performance of the predictive model developed using this data are highly encouraging. It is also a novel application for non-contact sensors in clinical settings, especially for HD patients. The authors found very few studies that used AI for the prediction of events in liver transplant patients, but could not find any study using AI on sensor data to predict clinical events in HD patients. On the basis of our findings, we recommend further studies using non-contact sensors to monitor the vital parameters of chronic kidney disease (CKD) patients during HD. This can be utilized to predict the possibility of medical problems in HD patients in advance. In addition, as a part of future work, we are further interested in analyzing this data more specifically using the available ICD code information reported during clinical events. This will enable us to identify whether there is any difference in the recordings when the event is reported as compared to when it is not reported. Furthermore, a patient might have reported several issues during the reporting of clinical events. It will be intriguing to learn whether there is any association between the different types of medical issues reported by the patients and the trends between different patients as far as the association of different medical issues are concerned. We also plan to check the importance of each feature variable used in the ML models. It will be intriguing to learn which feature variable has greater impact on the efficiency of the developed predictive ML models.

## Figures and Tables

**Table 1:** Baseline characteristics of the study data (*n* = number of patients).

Characteristics	Values (*n* = 109, All HD Sessions)	Values (*n* = 105, 15 HD Sessions)
Number of Male participants (%)	58 (53.2)	54 (51.4)
Number of Female participants (%)	51 (46.8)	51(48.6)
Age Range	30–89	30–89
Mean Age (±std.)	66.3 (±12.2)	66.4 (±12.2)
BMI Range	17.4–42.3	17.4–42.3
Mean BMI (±std.)	24.1 (± 3.6)	24.1 (±3.6)
Total Number of Hemodialysis Sessions	3237	1575
HD Sessions Range	7–52	15
Average No. of Sessions (±std.)	29.69 (±9.97)	15

HD: Hemodialysis; BMI: Body Mass Index.

**Table 2:** Details of events reported during study period.

Event Details	Values
Number of different events	5
Number of sessions with events	166
Number of patients reporting the event	78
Number of patients who did not report the event	31
Number of patients with sudden death	6
Number of patients reporting an ER visit	33
Number of patients reporting inpatient (IP)	32
Number of patients reporting ERIP	28
Number of patients reporting muscle spasm	45

ER: Emergency Room visit; ERIP: Emergency Room visit and Inpatient.

**Table 3:** Comparison of mean values of various parameters between the event and no-event class.

Features	Event (*n* = 11,400)		No Event (*n* = 4350)		*p* Values
	Mean	(SD)	Mean	(SD)	
HR_FFM	75.58	11.84	70.56	11.96	<0.0001
HR_LFM	75.86	12.33	70.62	12.01	<0.0001
RR_FFM	17.80	4.28	16.49	3.72	<0.0001
RR_LFM	17.50	4.35	16.12	3.49	<0.0001
HF_FFM	0.42	0.23	0.41	0.22	0.0035
HF_LFM	0.41	0.22	0.40	0.21	0.0803
LF_FFM	0.39	0.16	0.40	0.16	<0.0001
LF_LFM	0.39	0.15	0.40	0.15	<0.0001
LF/HF_FFM	1.30	1.16	1.36	1.23	0.0053
LF/HF_LFM	1.33	1.15	1.38	1.27	0.0187
VLF_FFM	0.35	0.19	0.35	0.17	0.0297
VLF_LFM	0.36	0.18	0.35	0.17	<0.0001
(VLF+LF)/HF_FFM	2.28	1.56	2.31	1.52	0.2177
(VLF+LF)/HF_LFM	2.34	1.55	2.32	1.51	0.4651
Age	66.18	12.45	67.10	11.33	<0.0001
BMI	24.14	3.91	24.32	2.78	0.0056

**Table 4:** Validation results of classifiers using stratified 10-fold cross-validation when data samples from all HD sessions were considered.

Model	Classifier	Mean Precision (±Std.)	Mean Recall (±Std.)	Mean Accuracy (±Std.)	Mean AUC (±Std.)
Model-1	AdaBoost	0.9407 (0.011)	0.8209 (0.015)	0.8381 (0.011)	0.8497 (0.013)
	kNN	0.9621 (0.004)	0.8847 (0.007)	0.8948 (0.004)	0.9016 (0.004)
	SVM	0.9019 (0.006)	0.7528 (0.011)	0.7694 (0.008)	0.7805 (0.007)
	RF	0.8352 (0.009)	0.6284 (0.014)	0.6528 (0.011)	0.6691 (0.012)
	LR	0.7908 (0.008)	0.5976 (0.008)	0.6073 (0.008)	0.6138 (0.009)
Model-2	AdaBoost	0.9047 (0.021)	0.8073 (0.026)	0.8051 (0.022)	0.8036 (0.026)
	kNN	0.8914 (0.019)	0.7178 (0.022)	0.7408 (0.019)	0.7562 (0.022)
	SVM	0.8711 (0.014)	0.7443 (0.025)	0.7436 (0.020)	0.7431 (0.020)
	RF	0.8665 (0.026)	0.6235 (0.047)	0.6688 (0.037)	0.6992 (0.035)
	LR	0.7928 (0.021)	0.6028 (0.038)	0.6114 (0.030)	0.6172 (0.028)
Model-3	AdaBoost	0.9417 (0.077)	0.8750 (0.125)	0.8618 (0.085)	0.8542 (0.101)
	kNN	0.8237 (0.136)	0.5875 (0.194)	0.6147 (0.165)	0.6354 (0.167)
	SVM	0.8821 (0.107)	0.6375 (0.221)	0.6594 (0.124)	0.6813 (0.109)
	RF	0.8639 (0.104)	0.8482 (0.093)	0.7897 (0.124)	0.7449 (0.167)
	LR	0.9437 (0.095)	0.8232 (0.139)	0.8362 (0.140)	0.8491 (0.155)

kNN: k-nearest neighbor; AdaBoost: adaptive boosting; LR: logistic regression; RF: random forest; SVM: support vector machine.

**Table 5:** Validation results of classifiers using stratified 10-fold cross-validation when data samples from the 15 recent HD sessions were considered.

Model	Classifier	Mean Precision (±Std.)	Mean Recall (±Std.)	Mean Accuracy (±Std.)	Mean AUC (±Std.)
Model-1	AdaBoost	0.9509 (0.012)	0.8186 (0.018)	0.8380 (0.016)	0.8538 (0.017)
	kNN	0.9635 (0.006)	0.8664 (0.010)	0.8795 (0.009)	0.8901 (0.009)
	SVM	0.8982 (0.008)	0.7622 (0.014)	0.7653 (0.012)	0.7679 (0.013)
	RF	0.8462 (0.021)	0.7196 (0.019)	0.7021 (0.022)	0.6879 (0.030)
	LR	0.8212 (0.012)	0.6216 (0.013)	0.6281 (0.014)	0.6334 (0.016)
Model-2	AdaBoost	0.8809 (0.023)	0.7886 (0.028)	0.7695 (0.027)	0.7541 (0.033)
	kNN	0.8889 (0.029)	0.7035 (0.042)	0.7213 (0.033)	0.7357 (0.036)
	SVM	0.8625 (0.043)	0.7404 (0.046)	0.7251 (0.042)	0.7127 (0.059)
	RF	0.8295 (0.038)	0.6982 (0.057)	0.6762 (0.039)	0.6583 (0.048)
	LR	0.8188 (0.046)	0.6202 (0.028)	0.6248 (0.042)	0.6285 (0.060)
Model-3	AdaBoost	0.7171 (0.094)	0.7464 (0.162)	0.6044 (0.143)	0.4899 (0.164)
	kNN	0.7733 (0.131)	0.4054 (0.136)	0.4824 (0.121)	0.5443 (0.127)
	SVM	0.7423 (0.117)	0.6071 (0.223)	0.5461 (0.139)	0.4869 (0.146)
	RF	0.6680 (0.143)	0.5393 (0.204)	0.4663 (0.152)	0.4113 (0.168)
	LR	0.7183 (0.073)	0.5893 (0.088)	0.5308 (0.069)	0.4780 (0.100)
